# Requirements for 5′dRP/AP lyase activity in Ku

**DOI:** 10.1093/nar/gku796

**Published:** 2014-09-08

**Authors:** Natasha T. Strande, Juan Carvajal-Garcia, Ryan A. Hallett, Crystal A. Waters, Steven A. Roberts, Christina Strom, Brian Kuhlman, Dale A. Ramsden

**Affiliations:** 1 Lineberger Comprehensive Cancer Center, Department of Biochemistry and Biophysics, University of North Carolina, Chapel Hill, NC 27514, USA; 2 Curriculum in Genetics and Molecular Biology, University of North Carolina, Chapel Hill, NC 27599, USA

## Abstract

The non-homologous end joining (NHEJ) pathway is used in diverse species to repair chromosome breaks, and is defined in part by a requirement for Ku. We previously demonstrated mammalian Ku has intrinsic 5′ deoxyribosephosphate (5′dRP) and apurinic/apyrimidinic (AP) lyase activity, and showed this activity is important for excising abasic site damage from ends. Here we employ systematic mutagenesis to clarify the protein requirements for this activity. We identify lysine 31 in the 70 kD subunit (Ku70 K31) as the primary candidate nucleophile required for catalysis, but additional mutation of Ku70 K160 and six other lysines within Ku80 were required to eliminate all activity. Ku from *Saccharomyces cerevisiae* also possesses 5′dRP/AP lyase activity, and robust activity was also reliant on lysines in Ku70 analogous to K31 and K160. By comparison, these lysines are not conserved in *Xenopus laevis* Ku, and Ku from this species has negligible activity. A role for residues flanking Ku70 K31 in expanding the range of abasic site contexts that can be used as substrate was also identified. Our results suggest an active site well located to provide the substrate specificity required for its biological role.

## INTRODUCTION

Double-strand breaks can be lethal if left unrepaired, and aberrant repair contributes to gross chromosome rearrangements. In many contexts the predominant DSB repair pathway in mammals is non-homologous end joining (NHEJ). This pathway mediates repair of DSBs using the core factors Ku, DNA-PKcs, XRCC4, DNA-Ligase IV and XLF. Absence of any of these core factors results in severe radiation sensitivity and immunodeficiency (among other phenotypes) (1). Among these core factors, Ku plays an essential role in end recognition, and additionally acts as a scaffold for stable assembly of a complex that then aligns ends together (2).

Ku as well as the other core factors also recruit additional enzymes to DSBs, including multiple nucleases, polymerases and specific end-cleaning enzymes like polynucleotide kinase-phosphatase. Processing enzymes are an important component of NHEJ because biological sources of DSBs possess complex end structures—ends with nucleotide damage, or when aligned have gaps or mispairs–that would otherwise block ligation (3). Abasic sites are one such class of nucleotide damage, and can be generated directly by ionizing radiation and radiomimetic drugs (4,5). Abasic sites are also a normal intermediate in base excision repair (BER), and if present on opposite strands and incised by apurinic/apyrimidinic (AP) endonuclease will generate a DSB with 5′ abasic site termini (aborted BER) (6–11). Such DSBs are also expected after attempted BER of the clustered oxidative damage associated with ionizing radiation, as well as during immunoglobulin class switch recombination (12–14).

Our group has shown that abasic site damage blocks ligation in cells, including by NHEJ, unless the abasic site can be excised by a deoxyribosephosphate (5′dRP) and AP lyase (also termed Class I AP endonuclease) (15). We also identified Ku as the principle source of 5′dRP/AP lyase activity in cell extracts in both rodent and human cell models, when the abasic site is near DSB termini. Additionally, a mutant in the Ku70 subunit (Ku70 3A) was identified that had reduced 5′dRP/AP lyase activity *in vitro*; when used to complement *Ku70^−/−^* dermal fibroblasts, this mutant had a corresponding reduced ability to promote end joining that was specific to ends with abasic site termini (separation of function mutation) (15). We thus confirmed a significant role for this activity in the context of cellular NHEJ.

**Figure 1.**
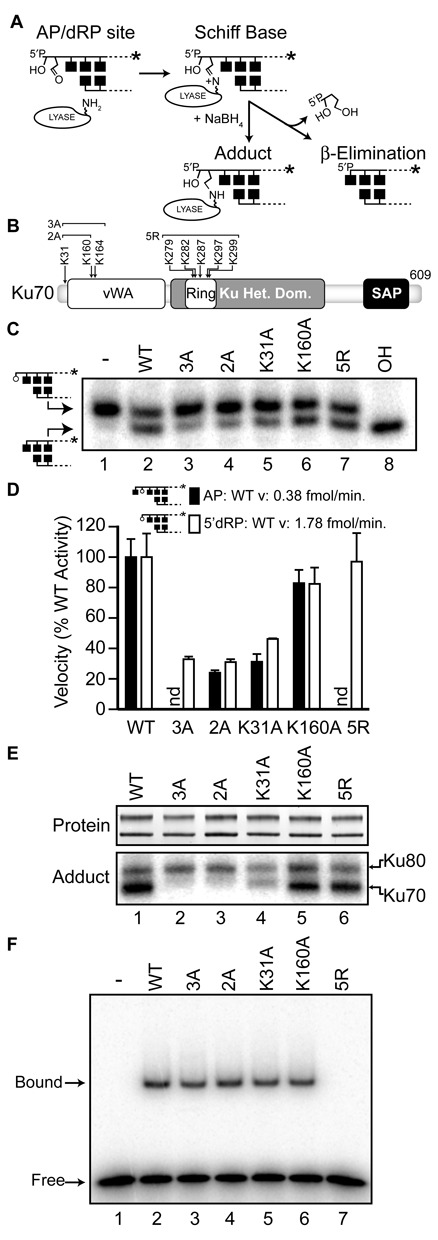
Identification of adducting lysines within human Ku70. **A.** Description of the 5′dRP/AP lyase reaction with abasic site, and use of NaBH_4_ to trap the covalent intermediate. **B.** Domain map of human Ku70 showing the location of lysines or groups of lysines that were mutated to alanine (A) or arginine (R). vWA, von Willebrand factor type A domain; SAP, SAF-A/B, Acinus and PIAS. **C.** Representative reactions were performed with 1 nM radiolabeled 5′dRP substrate with 5 nM purified recombinant Ku heterodimer at 37°C for 5 min, with the Ku70 subunit either WT or mutated as described in panel B. Reactions were analyzed by denaturing polyacrylamide gel electrophoresis (PAGE). In lane 8 substrate was treated with alkali (OH) to validate abasic site generation. **D.** Average lyase velocities were determined by incubating 5 nM purified recombinant Ku heterodimer and 1 nM AP (filled bar) or 5′dRP (open bar) substrates at 37°C, and expressed as a percentage of the velocity observed with WT heterodimer. Error bars are the standard deviation of triplicate determinations. **E.** Purified heterodimers were subjected to sodium dodecyl sulphate (SDS)-PAGE (SDS-PAGE) analysis and detected directly by SYPRO orange (top panel). Products of Schiff-base trapping assays (bottom panel) were detected by phosphorimaging after incubation of the heterodimer with radiolabeled 5′dRP substrate as in panel C, except reactions were supplemented with 5 mM NaBH_4_ and incubated for 10 min. **F.** EMSA was performed by incubating 1nM Ku with 1 nM radiolabeled 30 bp substrate for 15 min.

5′dRP/AP lyases cleave 3′ of abasic sites, and initiate cleavage by forming a Schiff base covalent intermediate between the enzyme's active site nucleophile and the abasic site 1′ carbon (Figure 1A), and this enzyme-substrate covalent intermediate can be trapped by reduction with NaBH_4_. Notably, Ku can be trapped to abasic site substrates generated through the action of a glycosylase as well as those generated by a radiomimetic (bleocin) (15). The active site nucleophile that forms the Schiff base is typically a lysine epsilon amino group, and many 5′dRP/AP lyases possess high levels of residual activity after mutation of the primary nucleophile through use of a different lysine located nearby. We therefore mutated candidate lysines in groups to identify a triple mutant (Ku70 3A) that ablated the ability of the Ku70 subunit to form a Schiff base (15), but the relative importance of individual residues for activity was not clear. Additionally, it was apparent even in the wild-type (WT) heterodimer that Ku80 also possessed at least one alternate nucleophile, which was subsequently shown to partly compensate for loss of Ku70 nucleophiles when in a heterodimer with the Ku70 3A mutant. Here, we systematically mutate lysines from both subunits of the human Ku heterodimer and identify the primary candidate nucleophile. We then use these results to investigate whether conservation of this amino acid is predictive of activity in other species. We additionally identify a role for residues flanking the primary nucleophile in expanding the range of substrates.

## MATERIALS AND METHODS

### Proteins and substrates

Constructs to express human Ku have been previously described (16). cDNAs for *Saccharomyces cerevisiae* Ku subunits were obtained from Dr. T. Paull (UT Austin), and a lysine at amino acid position 21 of Ku70 was reverted to threonine in accord with the Refseq record (NP_014011.1). We additionally removed the N-terminal hexahistidine tag on Ku70 as it interfered with DNA binding when in this location, and introduced this tag instead at the C-terminus as also arranged in the human Ku70 construct. cDNAs for *Xenopus laevis* Ku were obtained from Dr. H. Funabiki (Rockefeller University), and a C-terminal hexahistidine was introduced at the *X. laevis* Ku70 C-terminus as described for both *S. cerevisiae* and human Ku70. All constructs were then transferred to the Fastbac vector backbone for eventual overexpression using the Bac-to-Bac baculovirus expression system (Invitrogen), and purified after overexpression in insect cells as previously described (16). DNA-PKcs was extracted from HeLa cells and purified as described (17).

All substrates were made from gel-purified oligonucleotides (Integrated DNA Technologies). Deoxyuracil-containg strands were 3′ radiolabeled with terminal deoxynucleotidyl transferase and [α-^32^P] cordycepin (PerkinElmer). The 5′dRP substrate was made by annealing 5′P-UGGAAATCAAATGTAAGTAGAGGTCA-3′ with 5′biotin-Tetra-ethyleneglycol (TEG) -TTGACCTCTACTTACATTTGATTTC-3′. The AP substrate was made by annealing 5′P-GUGGAAATCAAACGTAAGTAGAATCCAAAGTCTCTTTCTTCCG-3′ to 5′biotin-TEG-TCGGAAGAAAGAGACTTTGGATTCTACTTACGTTTGATTTC-3′, and the alternate AP substrate used in Figure 5B and C made by substituting the top strand in the above AP substrate with 5′P-TTUGAAATCAAACGTAAGTAGAATCCAAAGTCTCTTTCTTCCG. A 30 bp substrate used for electrophoretic mobility shift analysis (EMSA) was made by annealing 5′P-GUGGAAATCAAACGTAAGTAGAATCCAAAGTCT-3′ to 5′biotin-TEG-TAGACTTTGGATTCTACTTACGTTTGATTTC-3′.

**Figure 2.**
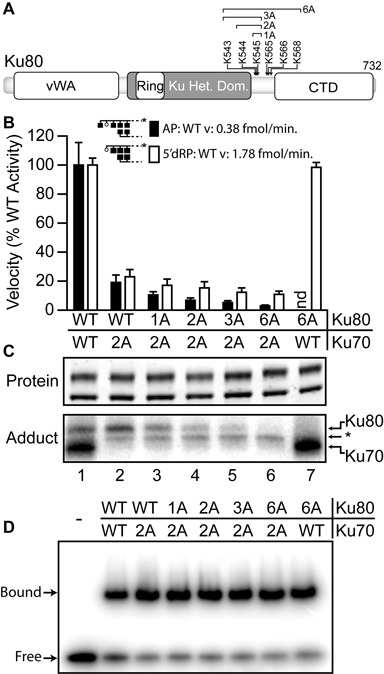
Identification of adducting lysines within human Ku80. **A.** Domain map of human Ku80 showing the location of lysines or groups of lysines that were mutated to alanine (A). **B.** Average velocities were determined from reactions at 37°C with 5 nM purified recombinant Ku heterodimer and 1 nM AP (filled bar) or 5′dRP (open bar) substrates, and expressed as a percentage of the velocity observed with WT heterodimer. Error bars are the standard deviation of triplicate determinations. **C.** Purified heterodimers were subjected to SDS-PAGE analysis and detected directly by SYPRO orange (top panel). Products of Schiff-base trapping assays (bottom panel) were detected by phosphorimaging after incubation of the heterodimer with radiolabeled 5′dRP substrate as in Figure 1, panel C, except reactions were supplemented with 5 mM NaBH_4_ and incubated for 10 min. *, unknown adducting species. **D.** EMSA was performed by incubating 1 nM Ku with 1 nM radiolabeled 30 bp substrate for 15 min.

**Figure 3.**
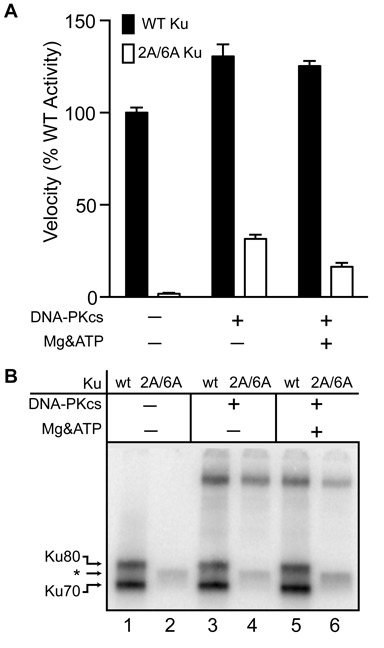
Effect of DNA-PKcs on 5′dRP/AP lyase activity. **A.** 1 nM radiolabeled AP substrate containing an abasic site was incubated at 37°C for 10 min with 2 nM Ku or the 2A/6A mutant Ku described in Figure 2. 2nM DNA-PKcs was included as noted. Mg&ATP, reactions were supplemented with 5mM MgCl_2_ and 100 μM ATP. Average velocities and standard deviations were determined from three independent experiments, and expressed relative to the activity observed with WT Ku heterodimer alone. **B.** Reactions were performed as in panel A, but supplemented with NaBH_4_, and analyzed by SDS-PAGE and phosphorimaging. *, unknown adducting species.

**Figure 4.**
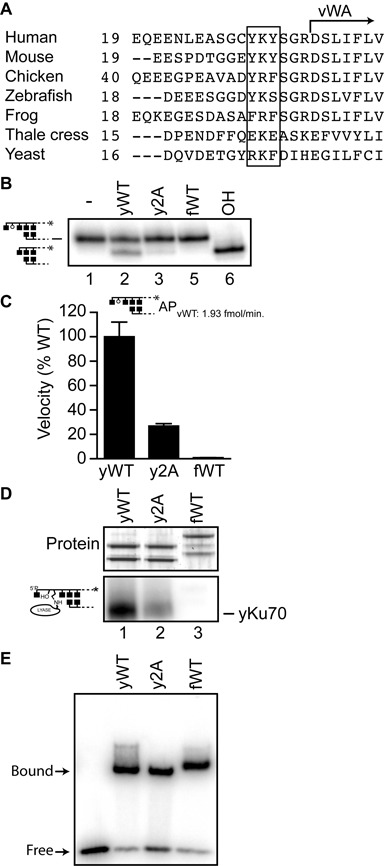
AP lyase activity of Ku from model organisms. **A.** Alignment of Ku70 from different species. The start of the vWA domain is noted. K31 and flanking aromatics (targeted for mutation in Figure 5) are boxed. **B.** Reactions were performed with 1 nM radiolabeled AP substrate with 5 nM purified recombinant Ku heterodimer at 37°C for 2.5 min, using recombinant Ku heterodimer from *S. cerevisiae* (yWT), a yKu heterodimer with K29A and K161A substitutions in Ku70 (y2A), or a recombinant Ku heterodimer from *X. laevis* (fWT). In lane 6, substrate was digested with alkali (OH) to validate abasic site generation. Reactions were analyzed by denaturing PAGE. **C.** Average velocities were determined from reactions at 37°C with 5 nM purified recombinant Ku heterodimer and 1 nM AP substrate, and expressed as a percentage of the velocity observed with WT yeast Ku heterodimer (yWT). Error bars are the standard deviation of triplicate determinations. **D.** Reactions were performed as in panel B, but supplemented with NaBH_4_, and analyzed after a 10 min incubation by SDS-PAGE and phosphorimaging. **E.** EMSA was performed by incubating 1nM Ku with 1 nM radiolabeled 30 bp substrate for 15 min.

**Figure 5.**
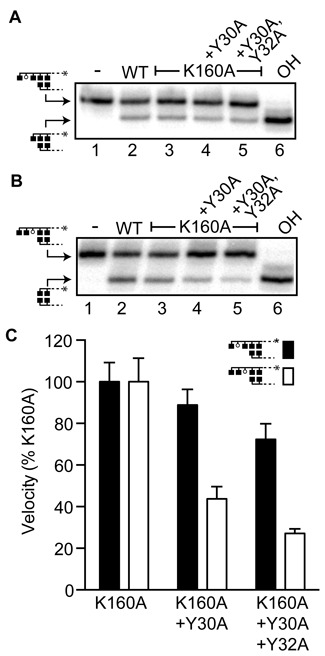
Function of aromatic residues flanking K31. **A** and **B.** Representative reactions performed with 5 nM WT human Ku heterodimers or Ku heterodimers with Ku70 K160A, Ku70 K160A and Y30A, or Ku70 K160A, Y30A and Y32A substitutions. **A.** Reactions used the standard AP substrate and were incubated for 15 min. **B.** Reactions used a variant substrate with an AP site located an additional nucleotide further from the 5′ terminus, relative to the substrate used in panel A (see cartoon), and were incubated for 30 min. **C.** Average velocities and standard deviations were determined from three independent experiments, and expressed relative to the average velocity observed with a Ku heterodimer containing Ku70 with the K160A substitution.

With the exception of reactions used for EMSA, annealed substrates were incubated with 50 nM streptavidin (Pierce) and 1 unit UDG (New England Biolabs) at 37°C for 5 min. Streptavidin was used to block the DNA end opposite of the 5′ dRP or abasic site, and thus confining Ku's ability to load onto the DNA in one orientation. This blocking was essential for accurate characterization of candidate nucleophiles; unblocked substrates, where Ku is also able to load on the substrate from the end distal to the abasic site (a biologically irrelevant substrate), had slightly reduced WT activity and the effects of mutations were reduced.

Sequences used in Figure 4A were from P12956 (*Homo sapiens*), P23475 (*Mus musculus*), O93257, (*Gallus gallus*), XP_005156194 (*Danio rerio*), Q9W626 (*X. laevis*), Q9FQ08 (*Arabidopsis thaliana*) and B3LMH2 (*S. cerevisiae*).

### Lyase reactions

Reactions were incubated in 25 mM NaPO_4_ pH 7.4, 0.1 mM ethylenediaminetetraacetic acid (EDTA), 125 mM KCl and 1 mM dithiothreitol at 37°C and terminated by the addition of 200 mM NaBH_4_ and an additional incubation on ice for 1 h. The products were analyzed by denaturing polyacrylamide gel electrophoresis and phosphorimaging (GE Biosciences). For velocity determination, aliquots were taken at three different times from triplicate reactions, with the duration of sampling increased for mutant proteins with very low activity to ensure the fraction of substrate that was converted to product was in a range that was accurately quantifiable (greater than 1%).

The covalent protein-DNA adducts were generated by assembly of reactions as above without Ku, and initiated by sequential addition of Ku and 5 mM NaBH_4_. Products were separated on a 4–12% Bis-Tris SDS polyacrylamide gel (Invitrogen) and analyzed by phosphorimaging (GE Biosciences).

EMSA analysis was performed by incubating 1 nM substrate with 1nM Ku for 15 min at 37°C, followed by electrophoresis on a 4% native polyacrylamide gel containing 1/3× TRIS/borate/EDTA buffer and analyzed by phosphorimaging (GE Biosciences).

### Modeling

We developed two PyRosetta (18) protocols to model the N-terminus of Ku70. The first protocol sampled only the torsion angles of the first eight residues of Ku70. The second protocol sampled backbone torsion angles of the first three nucleotides of the 5′ strand containing the abasic site as well as the first eight residues of Ku70. Both protocols used atom pair distance constraints between the amino nitrogen of K31 and the 1′ carbon of the abasic nucleotide. Moves to the peptide or DNA were evaluated by the Rosetta scorefunction and accepted or rejected according to a metropolis criterion (18). We evaluated final models by score and chose representative models for figures.

## RESULTS

### Identification of candidate primary nucleophiles in Ku70

Cleavage at abasic sites by 5′dRP/AP lyases is initiated by the nucleophilic attack of an amino group from the enzyme (often a lysine ϵ-amino) on the 1′ carbon of the abasic site (Figure 1A) (19). This generates a Schiff base covalent protein-DNA intermediate. This intermediate in turn promotes β elimination and cleavage 3′ of the abasic site, leaving behind a 3′ unsaturated sugar and 5′ phosphate as strand termini (Figure 1A). The covalent protein-DNA intermediate can be trapped by including the strong reducing agent, NaBH_4_, in the reaction, which converts the Schiff base to an amine, resulting in a stable enzyme-abasic site adduct (20). This assay was employed in previous work to show that while NaBH_4_ treatment trapped both the 70 and 80 kD subunits of Ku, Ku70 was the primary adducted species (15). We therefore initially mutagenized only the Ku70 subunit. We also focused on a specific region—the ‘ring’ through which DNA ends are threaded—that is, both the primary interface between Ku and DNA, as well as a rich source of lysines to act as nucleophiles (21) (Figure 1B). Combined substitution of five of the plausible candidate lysines from Ku70 in this ring to arginine (Ku70 5R) results in reduced affinity for DNA (Figure 1F). Nevertheless, when reactions are performed with Ku in excess over substrate, there is no significant impact of these mutations on 5′dRP/AP lyase activity (Figure 1C and D) or ability to form Ku70-DNA adducts (Figure 1E).

**Figure 6.**
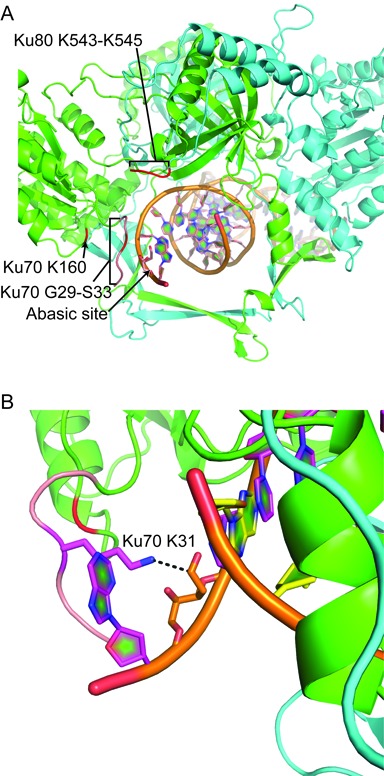
Model of the interaction between Ku70 K31 and an abasic site substrate. **A** and **B.** A cartoon representation of the human Ku heterodimer interacting with an abasic site substrate was modeled on the structure 1JEY (21). Five amino acids (pink) were appended to the most N-terminal residue resolved in Ku70 (G34), and the DNA altered to have an abasic site centered within a three nucleotide 5′ overhang. **A.** Ku70 is in green and Ku80 in cyan. The location of candidate nucleophiles identified in Figures 1 and 2 are shown in red. **B.** Ku70 K31 is shown in stick representation, with the dashed line showing the distance between nuclei predicted to participate in a Schiff base intermediate.

We therefore mutated additional groups of lysines in both the N-terminal vWA domain as well as the C-terminal portion of Ku70. As previously reported, only one such construct significantly impacted function, where three lysines within and immediately N-terminal to the Ku70 vWA domain (K31, K160 and K164) were substituted with alanine (Figure 1B; see also Figure 6A for location of these residues on the DNA-bound Ku structure) (15). The Ku70 3A mutation completely ablated ability of the Ku70 subunit to form a trapped intermediate, and resulted in reduced activity of the mutant heterodimer *in vitro* as well as reduced ability to promote NHEJ of abasic site-containing substrates in cells.

We assess here the contributions of each of these three lysines. Of the three lysines substituted in Ku70 3A, only two—K31 and K160—are located both near each other and DNA substrate. Accordingly, a mutant Ku70 with only K31 and K160 substituted (Ku70 2A) had activity indistinguishable from the previous Ku70 3A heterodimer (Figure 1C and D). Additionally, we could not trap Ku70 2A to abasic sites by addition of NaBH_4_, indicating K31 and K160, but not K164, are candidate nucleophiles and arguing they are the only significant candidates in this subunit (Figure 1E). Further analysis indicated that only the K31A substitution had a significant impact when mutated singly, reducing activity to 46 (±0.4)% of WT on a 5′ terminal abasic site (5′dRP), and 31 ± 5.0% of WT activity on an internal abasic site (AP) (Figure 1C and D). By comparison, the single K160A substitution had little impact; activity was reduced by this substitution primarily in the context of the K31A substitution. Moreover, the efficiency of adduction to Ku70 was reduced to a greater degree by substitution of K31 alone, when compared to K160 alone. Taken together, these results support a conclusion that Ku70 K31 is the primary nucleophile responsible for excision activity. Importantly, all aforementioned Ku mutants (with the exception of the Ku70 ring mutant) exhibit DNA binding indistinguishable from WT (Figure 1F).

Mutation of both candidate nucleophiles in Ku70 (K70 2A) still allows for significant activity: 31% when the abasic site is 5′ terminal (5′dRP), and 24% when the abasic site is not 5′ terminal (AP). Ku80 is the presumed source of this residual activity, as this subunit can be covalently adducted to the abasic site even in a WT heterodimer. The extent of Ku80 adduction also increases slightly in a complex with the Ku 70 2A mutation (Figure 1E).

### Characterization of residual activities in Ku80 and DNA-PKcs

We therefore investigated possible candidate nucleophiles in the Ku 80 subunit, focusing on a 26 amino acid segment from 543–568 bounded on each side by three lysines (Figure 2A). Nuclear magnetic resonance studies (22) and protease hypersensitivity (23) indicate this segment is a flexible linker between the central DNA binding domain and a C-terminal domain that interacts with DNA-PKcs, thus could plausibly locate near DNA and the previously characterized Ku70 2A pair. Importantly, substitution of all six residues in this linker blocked any ability of Ku80 to adduct to substrate, but had no impact on 5′dRP/AP lyase activity when in heterodimers with WT Ku70 (Figure 2B and C). However, activity was progressively reduced when the Ku70 2A subunit was combined with successive alanine substitutions in Ku80 of K545 (2A/1A), K544–545 (2A/2A), K543–545 (2A/3A) and finally all six lysines in Ku80 (K543–545 + K565, K566 and K568; 2A/6A). Each successive substitution resulted in only slight reductions in activity, but the cumulative effect (i.e. comparing the 2A/WT heterodimer to the 2A/6A mutant) was sufficient to reduce activity 2- to 5-fold, depending on whether 5′dRP or AP sites were used as substrate. These successive alanine substitutions in Ku80 also resulted in incremental reductions in ability to detect full length Ku80 adduct, eventually (i.e. using 2A/6A) resulting in undetectable levels of adduct formed to either full-length Ku subunit. The only remaining species is a contaminant intermediate in size between full-length Ku70 and Ku80 (*, Figure 2C). We have thus identified all significant candidate nucleophiles in human Ku. As with our Ku70 mutations, substitutions in Ku80 do not disrupt ability of mutant heterodimers to bind DNA (Figure 2D).

We previously reported that DNA-PKcs can also be adducted to abasic sites when included in reactions, but that activity of DNA-PKcs in the absence of Ku was very low (15). Here we show substitution of the 2A/6A mutant for WT Ku results in a 4- to 8-fold reduction in activity, depending on whether conditions permit kinase activity (Figure 3A). Ku thus remains the principle source of activity in reactions with both Ku and DNA-PKcs. Nevertheless, addition of DNA-PKcs to reactions with the 2A/6A mutant results in much greater activity than the very low level activity observed with the 2A/6A mutant alone. Adduction assays also identify a species of much higher molecular weight, relative to Ku subunits, consistent with identification of DNA-PKcs as a weak 5′dRP/AP lyase (Figure 3B).

### Activity of Ku orthologues

K31 in Ku70 is located five amino acids N-terminal to the beginning of the vWA domain (Figure 4A). A survey of Ku70 orthologs from model eukaryote organisms determined this candidate primary nucleophile is found in diverse species, including budding yeast (*S. cerevisiae*), Thale Cress (*Arabidopsis thaliana*), zebrafish (*Danio rerio*), mouse (*Mus musculus*) and human (*Homo sapiens*). Notably, this site is absent in Ku 70 from both frog (*X. laevis*) and chicken (*Gallus gallus*).

To assess if K31 is an accurate predictor of activity we focused on one species where it is present (*S. cerevisiae*; yKu), and one where it is missing (*X. laevis*; fKu). We expressed and purified recombinant ortholog heterodimers using the same methods previously used for generating recombinant human Ku. Activity was readily detected using yKu, while the *X. laevis* ortholog was inactive (Figure 4B and C). Activity of yKu was also reduced 4-fold upon substitution of K25 and K161 with alanine. Mutation of sites in yKu analogous to human K31 and K160 thus resulted in a loss in activity similar to that observed for the same mutation in human Ku. We were unable to detect significant levels of adduction to yKu80, or either subunit of the *X. laevis* heterodimer (Figure 4D). All three ortholog preparations (yKu, yKu 2A, fKu) were able to bind DNA (Figure 4E).

### Role of aromatic residues in expanding the range of potential substrates

Alignment of Ku70 orthologs also indicated one or both of the amino acids immediately flanking K31 are often aromatic (tyrosine or phenylalanine) (Figure 4A), and aromatic residues can have special significance to glycosylases and AP lyases (24–27). Since loss of K31-dependent activity is partly suppressed by presence of K160 we first made the K160A substitution, then in the background of this mutation substituted with alanine one or both of the tyrosines that flank K31 within human Ku70 (Y30, Y32). The resulting constructs—Ku70 K160A+Y30A, and Ku70 K160A+Y30A+Y32A—were made as heterodimers with WT Ku80, and activity compared to the Ku heterodimer with only the Ku70 K160A substitution.

Loss of both flanking tyrosines only slightly reduced the ability to excise abasic sites in the penultimate position (i.e. the standard AP substrate) (72, ±7.5%; Figure 5A and C). In contrast, these sites have a greater impact on activity when using a substrate with the abasic site located an additional nucleotide further from the 5′ terminus (Figure 5B and C). In this context, even the single Y30A substitution was sufficient to reduce activity ∼2-fold (44, ±7% of K160A). Strikingly, substitution of both tyrosines reduced activity to 27 ± 2.2%, relative to activity with the K160A substitution alone. This is comparable to the reduction expected if K31 were substituted instead. An adjacent aromatic residue is thus essential for K31 to act as a nucleophile, but only on a subset of substrates.

## DISCUSSION

We have clarified requirements for 5′dRP/AP lyase activity in Ku. We show K31, located five amino acids N-terminal to the vWA domain of human Ku70, is the only site that when mutated alone significantly impacts both activity and ability to trap intermediates. K31 can consequently be identified as the primary candidate nucleophile. Moreover, Ku from *S. cerevisiae* is active and this activity is largely dependent on lysines K24 and K161 (analogous to K31 and K160 in human Ku70). By comparison, these residues are not conserved in Ku from *X. laevis*, consistent with the absence of significant 5′dRP/AP lyase activity in Ku from this species. Whether Ku70 K31 is conserved or not in a given species thus appears sufficient to predict if Ku from that species possesses significant activity. Notably, when 5′dRP/AP lyase activity in human Ku was inactivated, the inclusion of DNA-PKcs provided some compensating activity. This suggests a mechanism for sustaining activity in vertebrate species where Ku is inactive.

The most N-terminal amino acid resolved for human Ku70 in the published crystal structure is G34 (PDB:1jey) (21), and density for K31 is therefore missing. To assess the feasibility of an interaction between human Ku70 K31 and substrate we appended five residues including K31 (G29-S33; pink in Figure 6A and B) to the N-terminus of the resolved structure. We then generated DNA similar to our *in vitro* substrates by positioning a ring-opened abasic site on the existing crystal structure DNA near the 5′ end, and also omitted three 3′ terminal nucleotides to generate a 5′ overhang. We modeled the complex using the PyRosetta molecular modeling suite (18). We were able to position the primary nucleophile within 4Å of the abasic site 1′ carbon (dashed line in Figure 6B), even without movement of the abasic site substrate (i.e. allowing movement only of the N-terminal extension; see below). The secondary nucleophiles for which there are available density—Ku70 K160 and Ku80 K543–545—were substantially farther away in all simulations. Identification of K31 as the primary nucleophile can thus be readily reconciled with the available structural information.

The existing crystal structure and mutagenesis data does not provide significant insight into other elements of substrate recognition. Most importantly, activity is severely inhibited by significant (greater than one base pair) double-stranded DNA 5′ of the abasic site (28). We suggest this is best explained by a need for bending of the DNA strand 3′ of the abasic site, as is observed in a model of the Pol β 5′dRP lyase active site (27). In addition to the restriction of activity to abasic sites within ssDNA, there are modest reductions (∼2-fold) in activity when the 5′ phosphate is missing, or if the 5′ phosphate is located more than two nucleotides 5′ of the abasic site (28). Bending of the overhang at the abasic site could thus allow for interaction between the 5′ phosphate and residues on the surface of the Ku70 vWA domain, and promote activity by helping fix the location of the abasic site relative to K31. Similarly, increased spacing between the 5′ phosphate and the abasic site makes activity reliant on tyrosines flanking K31, suggesting these residues help locate the abasic site when the distance between the abasic site and the 5′ phosphate is too great. Plausible mechanisms include a role for the aromatic residue in stacking between flanking bases (void-filling) (25,26), or in promoting the bending of the phosphate backbone 3′ of the abasic site (as with Y39 of Pol β) (27).

As noted and in accord with their differing biological roles, significant single-stranded character is required for activity in Ku (28) but inhibits Pol β activity (15,29). However, Pol β typically must act only on abasic sites immediately downstream of a nick, whereas substrate recognition by Ku must include abasic sites both terminal and near termini. Additionally, the location of Ku bound to DNA ends is not fixed (it can translocate internally). These differences may rationalize why the location of the primary nucleophile in Ku is in a region of the protein likely to be mobile, as well as the use of multiple strategies for fixing abasic site location relative to the nucleophile.
